# Serious sports-related injury in England and Wales from 2012-2017: a study protocol

**DOI:** 10.1186/s40621-020-00243-4

**Published:** 2020-04-27

**Authors:** Madeleine A. M. Davies, Tom Lawrence, Antoinette Edwards, Fiona Lecky, Carly D. McKay, Keith A. Stokes, Sean Williams

**Affiliations:** 1 Department for Health, University of Bath, Bath, UK; 2 Trauma Audit Research Network, Division of Population Health, Health Services Research and Primary Care, University of Manchester, Manchester, UK; 3 Centre for Urgent and Emergency Care Research, School of Health and Related Research, University of Sheffield, Sheffield, UK; 4 Centre for Sport, Exercise and Osteoarthritis Research Versus Arthritis, Nottingham, UK; 5 Rugby Football Union, Twickenham, UK

**Keywords:** Injury, Epidemiology, Sport, Trauma

## Abstract

**Background:**

Physical activity is an important component of healthy lifestyles, with a central role in morbidity prevention. However, sporting and physical activity also involve an inherent injury risk. Some sports and activities have a higher injury risk, and may involve more severe injuries. Furthermore, injuries of a severe nature have substantial individual and societal consequences, including the burden of assessment, treatment, and potential on-going care costs. There are limited data on severe sports injury risk in England and Wales, and no national data describing risk across sports. The aims of this study are to identify the cases and incidence of: i) paediatric and ii) adult severe sports injury from 2012 to 2017; and to describe injury incidence in individual sports.

**Methods:**

This study is an analysis of prospectively collected sport-related injuries, treated from January 2012 to December 2017. Incidents involving a severe injury (in-patient trauma care) in England and Wales, will be identified from the Trauma Audit Research Network registry. Data for patients who were: transfers or direct hospital admissions, with inpatient stays of ≥3 days, admissions to High Dependency areas, or in-hospital mortality after admission; and whose injury mechanism was sport, or incident description included one of 62 sporting activities, will be extracted. Data will be categorised by sport, and sports participation data will be derived from Sport England participation surveys. Descriptive statistics will be estimated for all demographic, incident, treatment and sport fields, and crude serious annual injury incidence proportions estimated. Poisson confidence intervals will be estimated for each sport and used to describe injury risk (incidence) across sporting activities.

**Discussion:**

This study will be the first to describe the number of, and trends in severe sport-related injuries in England and Wales. These data are useful to monitor the number and burden of severe sports injury, and inform injury prevention efforts. The monitoring and mitigation of sports injury risk is essential for individuals, health services and policy, and to encourage physically active lifestyles and safer participation for adults and children.

## Background

Sport and physical activity are associated with many health benefits. (Eime et al., 2013; The Department of Health, 2011; Warburton et al., 2006) However, participation also involves an inherent risk of injury. Injuries vary in severity from those which may involve pain but no treatment, to medical attention injuries not requiring removal from participation, to those requiring removal from participation and some time without sporting activity (i.e., time-loss injuries). Some injuries may involve surgical intervention and sustained functional limitation. Furthermore, some sports injuries may be career-ending, disabling, and potentially life-changing, such as those with spinal and cerebral involvement, often termed ‘catastrophic injuries’, (Bahr et al., 2020) and sport-related fatalities. As well as the symptomatic and functional consequences of injury for the individual, a greater severity of injury may be of greater societal consequence, with economic effects of injury, such as time lost from employment, potential time losst from employment for relatives and support providers, and an economic burden of injury assessment and treatment for health service providers. (Cumps et al., 2003; Kaplan et al., 2014; Ozturk & Kilic, 2013; Finch et al., 2015) In one American state over a 5-year period, youth sports injuries cost $24 million for in-patient care, $87 million for Emergency Department care, and an average of $6039 per inpatient visit. (Ryan et al., 2019) Meanwhile in the Netherlands, sports injuries had the third highest total (healthcare and productivity) costs, after home and leisure injuries, and traffic-related injuries. (Polinder et al., 2016)

The Trauma Audit Research Network (TARN) is a registry of traumatic injuries treated in hospitals in England and Wales, hosted at the University of Manchester with the coordination centre at Salford Royal NHS Trust. TARN coverage is national in England and Wales, and data are submitted by data collectors at hospitals, and approved for submission by coders at TARN. Case ascertainment within and between NHS trusts is monitored in comparison with eligible submissions according to Hospital Episode Statistics. TARN submissions are required as part of the Major Trauma Best Practice Tariff, and need to be met in order for payment to be approved. Inclusion criteria for an incident onto the TARN registry are: direct admissions or individuals transferred into hospitals for trauma-related care: of any age, who: i) stay in hospital for 3 days or more, ii) die after admission, or iii) are admitted to a high dependency area, High dependency facilities in British hospitals are those which provide more extensive care than a regular ward (such as higher staffing), and are equipped for an intermediate level of care, as often required after discharge from intensive care provision units. (Crosby, 2001) Additionally, potential cases need to meet isolated injury inclusion criteria, according to body region or injury, as outlined elsewhere. (Edwards et al.) Examples of isolated injury inclusion criteria (Table 1) are: head injuries (all brain or skull injuries, excluding injuries which are solely loss of consciousness, or injuries to the scalp). Data recorded for TARN-eligible incidents includes: date of admission, date of incident, geographic location of incident, incident location type (such as farm, public area, home, institution etc), time of admission, patient sex, patient date of birth, injury detail such as length, depth or grade of lacerations, fracture detail, blood loss, Abbreviate Injury Score, Injury Severity Score, diagnostic and treatment information, such as the use of CT scans and radiographs, Emergency Department observations and interventions, Operation details, outcome at discharge (alive, discharged to, readmission, Glasgow Outcome Scale), and rehabilitation prescription if applicable. These TARN-recorded injuries are by definition severe, representing those that require medical intervention and inpatient treatment, specialist treatment or high-dependency facilities.

**Table 1:** Isolated injury criteria for inclusion into the Trauma Audit Research Network (TARN) Registry. Reproduced with permission from Edwards et al. (b)

Body Region or Specific Injury	Included – in isolation^a^	Excluded – in isolation^a^
Head	All brain or skull injuries	Loss of consciousness or injuries to scalp
Thorax	All internal injuries	
Abdomen	All internal injuries	
Spine	Cord injury, fracture, dislocation or nerve root injury.	
Face	Fractures documented as: significantly displaced, open, compound or comminuted. All Le Fort fractures. All panfacial fractures. All orbital blowout fractures	Fractures documented as closed and simple or stable
Neck	Any organ injury, injury to the carotid artery, vertebral artery or jugular veins, hyoid fracture	Nerve injuries
		Skin injuries
Femoral fracture	All shaft, distal, head or subtrochanteric fractures, regardless of age.	Isolated neck of femur, inter or greater trochanteric fractures if > = 65 years.
	Isolated neck of femur, inter or greater trochanteric fractures if < 65 years old	
Foot or hand: joint or bone	Crush or amputation only.	Any fractures and/or dislocations, even if open and/or multiple.
Finger or toe	None	All injuries to digits, even if open
Limb – upper (except hands/fingers)	Any open injury.	Any closed unilateral injury (including multiple closed fractures and/or dislocations or the same limb)
	Any 2 limb fractures &/or dislocations.	
Limb – below knee (except feet/toes)	Any open injury.	including multiple closed fractures and/or dislocations or the same limb)
	Any 2 limb fractures and/or dislocations.	
Pelvis	All isolated fractures to ischium, sacrum, coccyx, ileum, acetabulum.	Single pubic rami fracture ≥65 years old.
	Single pubic rami fracture < 65 years old.	
	Multiple pubic rami fractures. Any fracture involving sacroiliac joint or symphysis pubis.	
Nerve	Any injury to sciatic, facial, femoral, cranial nerve or brachial plexus.	All other nerve injuries, single or multiple.
Vessel	All injuries to femoral, neck, facial, cranial, thoracic or abdominal vessels. Transection or major disruption of any other vessel (excluding vessels in the hands, feet and digits).	Intimal tear or superficial laceration or perforation to any limb vessel.
Skin	Laceration or penetrating skin injuries with blood loss > 20% (1000 ml).	Simple skin lacerations or penetrating injuries with blood loss <=20% (1000 ml); single or multiple. Contusions or abrasions: single or multiple.
	Major degloving injury (> = 50% body region).	
		Minor degloving injury (< 50% body region).
Burn	Any full thickness burn or partial/superficial burn > = 10% body surface area not referred to a burns unit.	Partial or superficial burn < 10% body surface area. Or any burn referred to a burns unit.
Inhalation	All included - if not referred to burns unit	If referred to burns unit.
Frostbite	Severe frostbite	Superficial frostbite
Asphyxia	All	None
Drowning	All	None
Explosion	All	None
Hypothermia	Accompanied by another TARN- eligible injury	Hypothermia in isolation
Electrical	All	None

^a^ Except where specified

There are tools to assist the classification of injury, such as the Abbreviated Injury Score, and Injury Severity Score, which indicate overall severity of injury for an individual. (Baker et al., 2002) In sport, there is international consensus on defining injury severity for a specific injury, and comparatively severe sports injuries are recognised to be those involving more than 28 days’ time-loss from sporting and physical activity (Bahr et al., 2020; Fuller et al., 2007; Hodgson et al., 2006), with no treatment parameters applied. This severity definition was derived from occupational injury time loss criteria. (Drawer & Fuller, 2002) However, despite this existing definition, and recognition of the important burden of comparably severe sporting injuries, there is no existing national routine severe injury monitoring in many countries, which would facilitate the identification of severe injury incidence, risk and mechanism in sport.

There is currently no national data on severe sports injury, severe sports injury risk, or sport-related catastrophic and fatal injuries in England and Wales. This data is needed to prioritise and develop adequate injury reduction strategies of injuries that occur during sport participation. (van Mechelen et al., 1992) Furthermore, an understanding of sport and physical activity injury permits informed physical activity decision-making for participants, can help support treating services and clinicians, and facilitate sports governing bodies in monitoring and mitigating injury risk. Therefore, this study aims to establish the number, type, and risk (cumulative incidence) of severe and catastrophic sports injuries presenting to English and Welsh healthcare services, for adults and children. Severity is being defined as an incident involving in-patient trauma care, and TARN-registry inclusion. This project will be the first to nationally estimate the incidence of severe and catastrophic sports injuries for i) adults and ii) children, and in identifying the burden and potential risk factors for severe injury, begin to work towards national safety promotion (Hanson et al., 2012) in sport.

## Objectives

The specific objectives of the first phase of this project will be:

1. To identify all NHS-treated severe sport-related injuries in England and Wales between 2012 and 2017.
2. To determine the cumulative incidence of severe injury across British highest participation (Active Lives Survey) sporting activities.
3. To describe the cumulative incidence and characteristics of severe injury per year from 2012 to 2017, in the highest national participation sporting activities (Active Lives Surveys), for i) Adults and ii) Children
4. To determine the proportion of all sports injuries that are classified as catastrophic (Injury Severity Score > 15), and the proportion of incidents which are fatal

## Methods

### Study design

This study will be a population-based cohort study of prospectively collected severe sporting trauma incidents, treated in the NHS and recorded in the TARN registry. The primary outcome will be any TARN-eligible sport-related injury. Every TARN submission is reviewed and approved by TARN coders, in addition to hospital-level performance comparisons, which are published by TARN every 4 months, and show data accreditation (complete fields), and case ascertainment of eligible incidents in each hospital and Trust compared to those potentially eligible, as indicated by Hospital Episode Statistics. (Edwards et al., 2018b) The case ascertainment for the study period between January 2012 and December 2017 was 79.5%.

### Case definition

Data for any sports injury qualifying for inclusion within the TARN registry, (Edwards et al.) occurring between January 2012 and December 2017 will be extracted. Eligible incidents will be those qualifying as TARN cases, (Edwards et al.) and indicated as occurring in sport or featuring one of 62 sporting activities in the free-text incident description field (Table 2). There will be no exclusion criteria based on age, or any other characteristics.

**Table 2:** Search terms for the free-text search of the incident description field of the Trauma Audit Research Network registry and their corresponding categorisation within the Active Lives Survey (November 2015 onwards)

Search term	Included as specific activity	Broad activity group	Composite activity groupings
American football		Sporting Activities	Team sports (other)
Athletics	Track and field athletics	Sporting Activities	Running, athletics or multi-sports
Badminton	Yes	Sporting Activities	Racket sports
Basketball	Yes (excluding wheelchair basketball)	Sporting Activities	Team sports
Bouldering	Climbing	Sporting Activities	Adventure sports
Boxing	Yes	Sporting Activities	Combat sports, Martial Arts or Target Sports
Canoe		Sporting Activities	Water sports
Cheerleading		Sporting Activities	Gymnastics, trampolining or cheerleading
Class	–	–	–
Climbing	Yes – hillwalking, hiking, rock climbing and bouldering	Sporting Activities	Adventure sports
Competition	–	–	–
Cricket	Yes	Sporting Activities	Team sports
Equestrian	Yes	Sporting Activities	Horse riding
Exercise	–	–	–
Fencing		Sporting Activities	Leisure games and activities
Field hockey		Sporting Activities	Team sports
Football	Yes	Sporting Activities	Team sports
Gaelic	Not unless reported as football	Sporting Activities	Team sports
Gokarting		Sporting Activities	Motorsports
Golf	Yes	Sporting Activities	Golf
Gym		Fitness Activities	Weights session or interval session
Gymnastics	Yes	Sporting Activities	Gymnastics, trampolining or cheerleading
Horse racing	Equestrian	Sporting Activities	Horseriding
Horse riding	Equestrian	Sporting Activities	Horseriding
Ice hockey	No	Sporting Activities	Winter sports
Ice skating	No	Sporting Activities	Winter sports
Jockey	Equestrian	Sporting Activities	Horseriding
Judo		Sporting Activities	Combat sports, Martial Arts or Target Sports
Karate		Sporting Activities	Combat sports, Martial Arts or Target Sports
Karting		Sporting Activities	Motorsports
Kayak		Sporting Activities	Water sports
Lacrosse		Sporting Activities	Team sports
Martial arts		Sporting Activities	Combat sports, Martial Arts or Target Sports
Motocross		Sporting Activities	Motorsports
Netball	Yes	Sporting Activities	Team sports
Obstacle course		Sporting Activities	Running, athletics or multi-sports
Olympic lifting		Fitness Activities	Weights session
Rat Race		Sporting Activities	Running, athletics or multi-sports
Roller derby		?	? Roller or skating sports
Roller skating		Sporting Activities	Roller or skating sports
Rowing	Yes	Sporting Activities	Water sports
Rugby		Sporting Activities	Team sports
Rugby league		Sporting Activities	Team sports
Rugby union	Yes	Sporting Activities	Team sports
Running	Yes	Sporting Activities	Running, athletics or multi-sports
Sailing		Sporting Activities	Water sports
Skate boarding		Sporting Activities	Roller or skating sports
Skating		Sporting Activities	Roller or skating sports
Skiing	Snowsports	Sporting Activities	Winter sports
Snowsports	Yes	Sporting Activities	Winter sports
Spartan Run		Sporting Activities	Running, athletics or multi-sports
Squash	Yes	Sporting Activities	Racket sports
Swimming	Yes	Sporting Activities	Swimming, diving or water polo
Taekwando		Sporting Activities	Combat sports, Martial Arts or Target Sports
Tennis	Yes	Sporting Activities	Racket sports
Tough Mudder		Sporting Activities	Running, athletics or multi-sports
Tournament		–	–
Training		–	–
Trampoline		Sporting Activities	Gymnastics, trampolining or cheerleading
Trampolining		Sporting Activities	Gymnastics, trampolining or cheerleading
Triathlon		Sporting Activities	Running, athletics or multi-sports
Weight		Fitness Activities	Weights session
Weight lifting		Fitness Activities	Weights session
Weight training		Fitness Activities	Weights session
Wheelchair basketball		Sporting Activities	Team sports
Wheelchair rugby		Sporting Activities	Team sports
Wrestling		Sporting Activities	Combat sports, Martial Arts or Target Sports

### Exposure estimation

The Sport England Active People, and Active Lives Surveys provide an annual estimate of sporting and physical activity in England. The Active People Survey was replaced by the Active Lives Surveys from 2015. There are different surveys for adults (≥16 years), and children and young people. (Sport England, 2017a) The Active Lives Adult Survey selects a random sample of households in England from the Royal Mail’s Postal Address File, and invites individuals at those addresses to complete a questionnaire regarding their sporting activities, habits and spectatorship. (Sport England, 2017b) The Active Lives Adult Survey is bi-annual, and participation data for the denominator will be from the composite activity groupings (Table 2), of the Adult Active Lives Adult Survey. Data collection years 2 and 3, covering the period from November 2016 until November 2018 will be used. This denominator data period does not encompass the entire study period, however the variables of interest which provide the best estimate of national sports participation to date were collected from Year 2 onwards. Discussions with Sport England and the study’s oversight committee have established that using Years 2 and 3 together as an estimate of annual sports participation will provide the best available estimate of national sports participation. In Year 2, there was a 18.9% response rate to the questionnaire, and 198,911 participants. (Mori, 2018a; Sport England, 2019) Twice-monthly participation (has taken part at least twice in the last 28 days, for selected activity groups) for sport will be used to indicate sports participation, and be used as denominator data.

The Active Lives Children and Young People Survey is annual and schools-based, with schools randomly selected from the Edubase register of educational establishments in England. Up to 3 year groups are randomly selected for a participating school, and within these year groups, a single mixed-ability class is randomly selected, therefore contributing up to three classes per school. The Active Lives Children and Young Peoples Survey started in this format in September 2017, and includes a sample of children from school Year 1 (ages 5 and 6), through to Year 11 (ages 15 and 16), across the academic year, from the autumn, spring or summer terms. (Mori, 2018b) Due to these differences in exposure data collection between adult and paediatric sports participants, and the comparatively more difficult identification of in-game sport for paediatric participants (i.e. ‘running around’, ‘kicking football in park’), it is envisaged the results for each of these populations will be presented separately.

### Ethical considerations and permissions

University of Bath Research Ethics Approval Committee for Health (REACH) favourable opinion has been granted for this project (REACH: EP 17/18 08), and a Research Request Form detailing the study’s title, research question and hypothesis has been approved by TARN’s Research Committee (170703). A Data Transfer Agreement has been established between the Universities of Bath and Manchester (hosting TARN data, with the coordination centre at Salford Royal hospital), to ensure acceptable data management and security processes.

### Data management and oversight

Anonymised data will be securely transferred by Data Management staff at TARN using ZendTo software and stored on password-protected and managed research storage at the University of Bath. All received data will be checked for accuracy and outliers, in addition to the routine data monitoring of TARN data undertaken by TARN, for both data accreditation, and case ascertainment. (Edwards et al.)

An independently-chaired project steering group (membership *n* = 13), has been established involving key project stakeholders across injury and trauma management, sport and exercise medicine, members of the public with experience of severe traumatic sporting injuries, TARN registry experts, policy experts, sports injury researchers, and other project stakeholders. The purpose of this group is to help direct the project, inform its plans, progress and accountabilities, and to ensure the project remains to schedule, is widely disseminated to appropriate audiences, and is accountable to patients and people with severe sporting injuries. Any conflicts of interest in the steering group have been declared at initial meetings, and are re-stated and documented at subsequent meetings. A quorum has been identified and agreed for consensus decisions within this steering group (minimum of 8 members present).

### Data cleaning and validation

Data will be categorised according to sport and physical activity undertaken at the time of the incident, and any non-sporting or physical activity injuries will be excluded. Using Stata 13.1 (StataCorp, Texas), a sporting indicator variable will be generated by one coder, for each of the 67 key phrases representing 62 sport or physical activities (Table 2), searched in the free-text incident description field, using the ‘foreach’ command to extract a potential case. Case extraction will be undertaken by searching for key words and their synonyms in the incident description field.

Each potential case will be flagged, and the incident description field then manually reviewed by the same coder, to detect any non-cases, which will be recoded. The confirmed cases and suspected non-cases will each then be manually reviewed a second time to ensure correct classification. This will be undertaken for each sport individually, and then the logic consistency checked between sporting activity categories.

Incidents remaining unclassified when all a priori sports are categorised will be individually reviewed, and either listed as a non-sporting incident or added to a data-driven list of new sporting categories activities. If an unclassified incident should be included within an existing category, search criteria for flagging the sport will be modified where appropriate to ensure inclusion. This will also be applicable for frequent misspellings, which will be identified from those incidents remaining unclassified, and then the search terms revised, and code re-ran. Incidents classified as non-sporting will be excluded on an individual basis using the entirety of their incident description field. New categories will then be generated for the data-driven list of sporting activities recorded in TARN data. To validate the categorisation process, a second researcher (SW) will independently classify a randomly generated subgroup of 10% of incidents to sporting groups or non-sporting activity, and the agreement between researchers will be assessed. If there are low counts of any individual sport, it is planned that these will be presented descriptively, but not carried forwards for incidence analysis. There is also the potential to group sports by Sport England’s Composite Activity Grouping of activities (Table 2), if there are low counts across sports. Patient anonymity will also need to be considered where there are fewer counts, in order to avoid presenting data on a rare scenario, which may therefore become identifiable. The study’s independently-chaired project steering group will additionally review all project decision-making and discuss any queries on inclusion of incidents identified during data cleaning.

### Statistical methods

Descriptive statistics (mean ± standard deviation for continuous variables, and number [percentage] for categorical variables) will be estimated for all demographic, incident, location type, treatment, and derived sporting activity fields. Crude cumulative serious injury incidence (proportions) will be estimated by dividing the mean annual number of serious injuries per year over the 6 year period, by the number of people participating in overall sporting activity per year, for 2012, 2013, 2014, 2015, 2016 and 2017 These data will be reported as i) the rate of severe injury per 100,000 participants per year, and ii) the rate of severe injury per 100,000 participants for each sporting activity.

Poisson confidence intervals will be estimated for each sport and physical activity, and used to describe severe injury rate in sporting activities. The proportion of catastrophic injuries (Injury Severity Score > 15) (Baker et al., 1974), and fatalities once reaching hospital, relative to all injuries, will be reported. Poisson confidence intervals will be used to assess the influence of injury risk factors (such as age and sex), on severe injury incidence.

### Power calculation

Due to the lack of published data for severe sports injuries nationally, it is difficult to provide accurate sample size justifications. However, a recent study identified that 18.3% (*n* = 63,877) of attendances to Emergency Departments in one English county were sport-related, and using regional TARN data from one county in 0–19 year olds, 324 sport or recreational activity related admissions were recorded during this 36-month period. (Kirkwood et al., 2019) Bahr et al. (2003) have suggested that in order to detect moderate to strong associations of a risk factor on injury risk, 20–50 injury cases would be required. (Bahr & Holme, 2003) During a 36-month period in one county, there were 91 sport and recreational injury-related incidents in one county, in 0–19 year olds. If this 3 year period (January 2012 to January 2015) were to be typical of our 5-year period, there would be 151.6 injuries per county in a 5-year period, and for across the 48 counties in England would provide 7276 cases in 0–19 year olds. If there were to be injury cases for each of the 62 sports used as search terms for this study, according to Bahr et al., we would require between 1240 and 3100 injured cases for our 62 sports to demonstrate moderate to strong associations of a risk factor on injury risk. Small to moderate associations would require approximately 200 injured cases, which would be 12,400 cases for our 62 sports. As there are approximately 7276 cases in 0–19 year olds, across England and Wales, and in all age groups, we should be adequately powered to describe the injured samples in each sport, and compare the incidence proportion between sports.

## Dissemination and knowledge translation

It is envisaged that study findings will be presented at academic and non-academic conferences and meetings to sports medicine, sports science, and healthcare professional and governmental audiences. Study findings will be formally reported to the project steering group and made available to interested parties. The project’s steering group has sought to be inclusive, independently chaired, and involve researchers alongside individuals with a history of severe sports injuries who can bring their experiences and knowledge to the project, (Davies et al., 2017; INVOLVE, 2012) in addition to knowledge users and stakeholders who may have the capacity to affect change, given the study’s findings. This is, to our knowledge, the first time that integrated knowledge translation (IKT), (Kothari et al., 2017) has been reported from the outset of sport-related research in England. Study findings will also be disseminated in scientific journal articles, through the University of Bath, and in community knowledge exchange and outreach activities.

## Discussion

There has been a move to identify the burden of sport and exercise injuries, with a view to promoting safer participation in physical activity for participants of all ages. Furthermore, where physical and sporting activities are encouraged as a public health intervention to decrease obesity and comorbidity, it is important to have an understanding of potential associated risks, and identify those at risk, with a view to injury prevention and health protection. This study will be the first to identify the number and type of severe sport and physical activity injuries treated in the NHS in England and Wales. It will also be the first to estimate the proportion and characteristics of severe sport injury, using national datasets. Furthermore, the risk of severe injury will be identified for both adult and child participants. Undertaking this novel national project of sport and physical activity injury risk begins to identify the health service burden associated with participation and sport and physical activity.

Strengths of this study including using a national dataset which is reflective of the clinical burden of severe sports injury, with good data completeness and case ascertainment. The study will also identify injuries from 62 prominent sporting activities, alongside those indicated as occurring during sport. This will enable many sporting activities to be captured and will not rely on all incidents being coded by TARN as occurring in sport, nor dictate which sports are exclusively featured in analysis. Potential weaknesses of this study include that not all sport-related mortality will be accounted for, as patients will need to reach and be admitted to hospital in order to be eligible for TARN registry inclusion. To address this, alternative data sources will be sought to identify cases of sport-related mortality. Additionally, requiring incidents to reach hospital will mandate that these injuries are particularly severe in nature, as they require patient care and observation or intervention over several days in hospital, as opposed to the routine definition of > 28 days timeloss from sport or physical activity, used in many other sport-related surveillance studies. (Bahr et al., 2020) We will not be able to determine the length of absence from sporting any physical activity as a measure of severity in this study, but will have data on the length of stay, and length of stay in critical care facilities, if applicable.

It is acknowledged that the study period for the denominator data does not encompass the entire period of TARN data extraction. However, Active Lives Adults Survey collects sports participation with the level of granularity required in terms of individual and team sports for this analysis, and specific variables required were only collected from Year 2 onwards. There is also caution exercised by Sport England on the interpretation of any year-on-year increases or decreases in participation, which may be resultant of survey’s sampling strategy more so than a true increase or decrease in participation. Therefore 2 years of Active Lives Adult Survey data were used, to lower the effect of denominator data sampling strategy on sports injury risk estimates. There may be a limitation in the TARN registry satisfactorily capturing paediatric injury, as the phraseology of childrens’ behaviours (i.e. was running when fell; kicking football and tripped over), may not be as reflective of coordinated sporting activity as for adult participants. Until this data has been retrieved and reviewed, this cannot be confirmed, and therefore it is planned that these groups will both be analysed, and analysed separately. However, our confidence in this paediatric data for identifying true in-sport injury, will be reviewed when the free-text injuries are categorised, and not presented in the main manuscript if it is not felt to be reflective of true in-sport activity.

Despite there being surveillance systems for sport-related severe injuries and fatalities, and research in this area in other countries such as the United States and Australia, (Endres et al., 2019; Kucera et al., 2019; Fortington et al., 2017; Dompier et al., 2019; Ekegren et al., 2018) there are currently no national estimates of sports injury risk in England and Wales. This endeavour will therefore greatly strengthen the literature in this field in England and Wales, and identify similarities and differences in severe injury incidence globally. Furthermore, in examining sports injury risk over 6 years from 2012 to 17, this study will be able to elucidate changes in sport and physical activity participation, and changes in sport and physical activity injury risk, over time. Recent studies in Australia have reported that the incidence of sport and recreation related hospital admissions are stable nationally with an annual incidence of 281.0 per 100,000 population between 2002 and 2012, (Lystad et al., 2019) however non-fatal major trauma may be increasing in individual states, with an increase in Victoria from 7.5 cases per 100,000 participants in 2005–6, to 14.01 injuries per 100,000 participants in 2014–15. (Ekegren et al., 2018; Andrew et al., 2012) In the US, sport-related traumatic brain injuries treated in Emergency Departments have also increased. (Coronado et al., 2015)

Undertaking a national study from a dataset such as TARN and using all data suggested as occurring during sporting activity will also support the identification of severe injury cases that may be occurring in sports with less resource and central coordination. These may also be sports that have historically not been able to participate or engage in medical and health-related research, due to limited resource. Some of these sports, which are not professional in the UK or have lower levels of participation, such as roller derby, American football or motorsports, may additionally present with unique trauma scenarios, and identifying these cases in one resource will facilitate further research around clinical management education, event management strategies, or adverse events.

This study aims to elucidate the number, type, and risk of severe sport and physical activity injuries in England and Wales. It is hoped that this data will identify intervention opportunities, and begin to identify the treatment pathways, characteristics, and burden of these injuries. This data will be relevant to clinicians, healthcare professionals, sports governing bodies, parents and organisations associated with sport and physical activity participation nationally in England and Wales. By engaging national stakeholders in the project, it is hoped that this project will be the start of a concerted effort to collaboratively monitor, evaluate and reduce severe sport and physical activity injury nationally.

## Data Availability

Data sharing is not applicable to this article as no datasets were generated or analysed during the current (protocol) process.

## Abbreviations

TARN: The Trauma Audit Research Network
NHS: The National Health Service
REACH: Research Ethics Committee for Health
